# A Curious Case of Pseudothrombocytopenia due to *In Vitro* Agglutination

**DOI:** 10.1155/2020/6236350

**Published:** 2020-02-13

**Authors:** Le Zhong, Juskaran Chadha, Ali Ameri

**Affiliations:** Department of Medicine, Zucker School of Medicine at Hofstra/Northwell at Lenox Hill Hospital, 100 E 77th St., New York, NY 10075, USA

## Abstract

Pseudothrombocytopenia (PTCP) is a laboratory phenomenon that can occur in hospitalized patients, with approximately 0.1 to 0.2% due to ethylenediaminetetraacetic acid (ETDA). The EDTA-dependent mechanism of PTCP occurs due to a conformational change of platelet surface glycoprotein IIb/IIIa (GPIIb/IIIa) caused by EDTA, which allows natural IgM or IgG auto-antibodies to bind to GPIIb/IIIa, leading to platelet agglutination. In most cases, PTCP resolves when a repeat blood sample is drawn in collection tubes containing either citrate or heparin. Here, we report a case of a 23-year-old female presenting with symptoms of gastroenteritis. She exhibited PTCP with blood draws obtained in not only collection tubes containing ETDA, but also with collection tubes containing heparin and citrate, which is highly unusual. The lack of resolution of platelet clumping in collection tubes containing either heparin or citrate suggests that heparin or citrate may also cause conformational changes to platelet surface glycoproteins in a similar mechanism as that of EDTA that allows binding of certain auto-antibodies.

## 1. Introduction and Case Presentation

A 23-year-old female with history of childhood asthma and eczema presented to the hospital's emergency department (ED) with 4 hours of epigastric abdominal pain, vomiting, and diarrhea. Review of systems was negative for fevers, chills, or any signs or symptoms of bleeding. Vital signs were stable on arrival. Examination revealed no signs of bleeding, including no rashes, petechia, purpura, or ecchymoses on skin examination.

Laboratory studies were obtained in the ED, including a blood draw obtained in a collection tube containing ethylenediaminetetraacetic acid (EDTA) obtained via venipuncture and analyzed for a complete blood count (CBC) using a Sysmex XN-10 CBC analyzer (Table 1). Notably, the initial CBC revealed a platelet count of 11 × 10^9^/L; WBC count and hemoglobin were within the reference ranges. Repeat CBC obtained in EDTA later the same day after admission to the hospital showed platelet count of 10 × 10^9^/L. Given the initially reported thrombocytopenia to 11 × 10^9^/L in the absence of any significant hematological history, the initial clinical concern was for idiopathic thrombocytopenic purpura (ITP) and the patient was treated with one dose of oral dexamethasone 40 mg.

**Figure 1:**
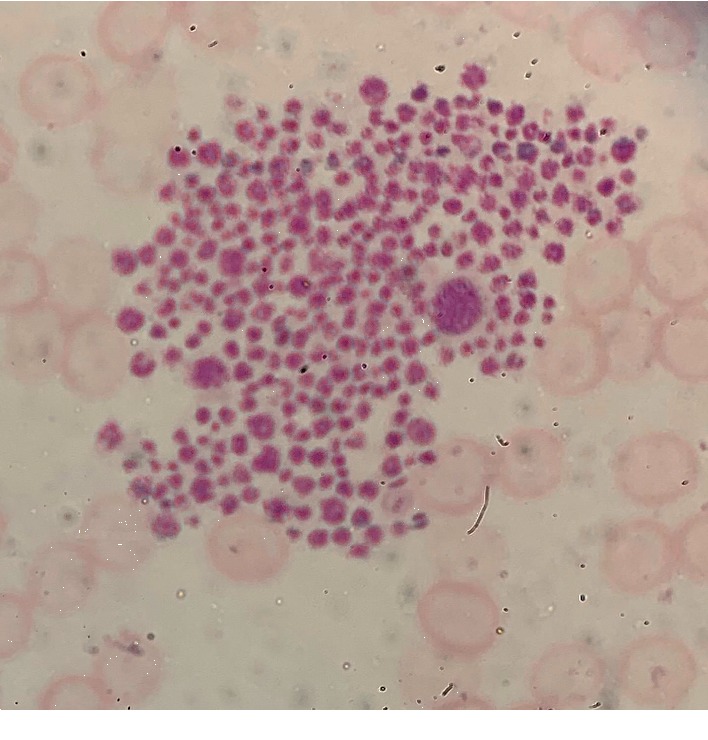
Peripheral smear of CBC drawn in tube containing EDTA (100X/1.30 oil microscope).

Given the finding of severe thrombocytopenia without signs or symptoms of mucocutaneous bleeding, alternate etiologies of the patient's thrombocytopenia besides ITP were suspected and a peripheral blood smear was prepared from the blood draw sample obtained in EDTA. Examination of a peripheral smear of blood samples collected into tube containing EDTA on Day 1 revealed significant platelet clumping (Figure 1). On the peripheral smear, platelet clumping can be found by examining the smear under 40X magnification under a oil light microscope until clusters of platelets can be visualized. The magnification can then be increased to 100X while centering the cluster of platelets to further examine clusters of platelets to identify platelet clumping.

**Figure 2:**
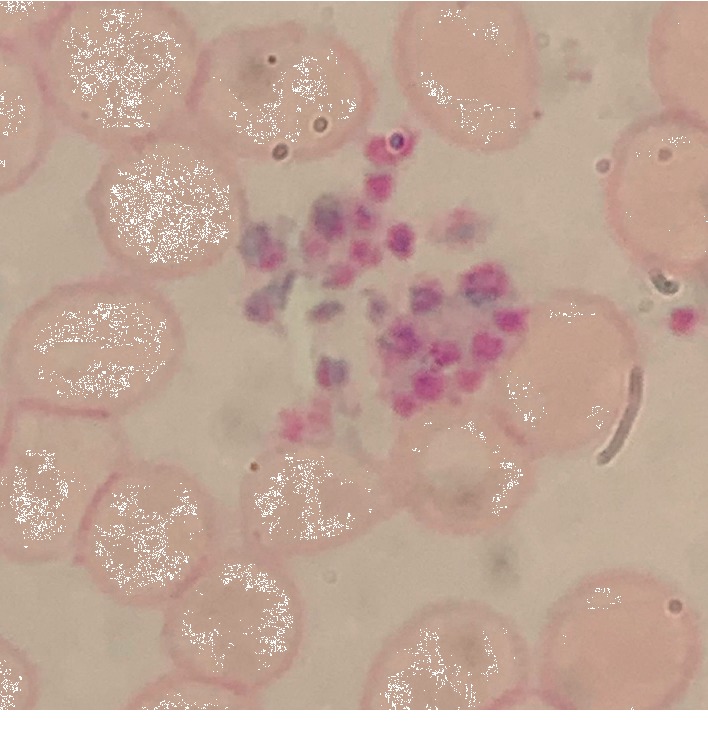
Peripheral smear of CBC drawn in tube containing heparin (100X/1.30 oil microscope).

**Figure 3:**
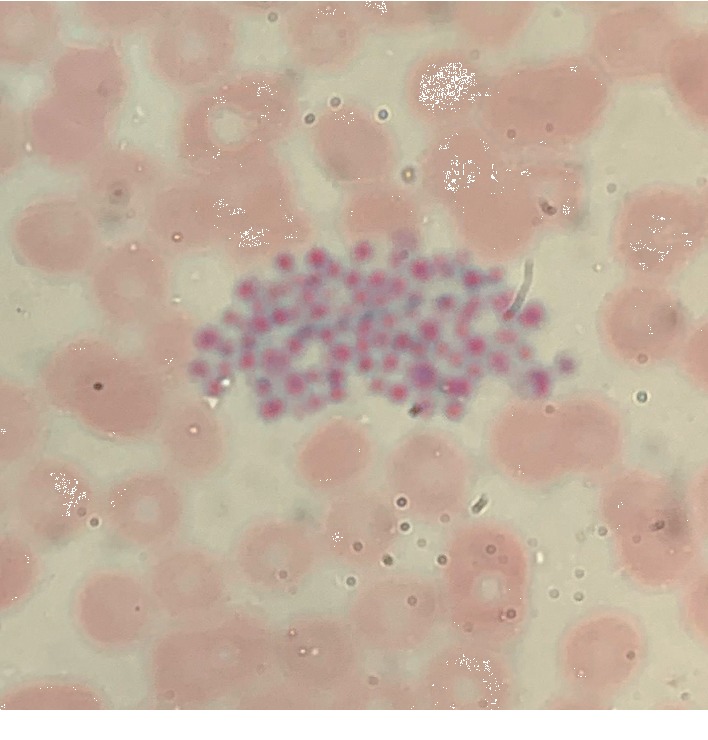
Peripheral smear of CBC drawn in tube containing citrate (100X/1.30 oil microscope).

On the morning of Day 2 of admission, a CBC obtained via EDTA collection tube showed platelet count of 10 × 10^9^/L, while blood collection at the same time obtained in a tube containing citrate showed a platelet count of 53 × 10^9^/L. Repeat blood collection with tube containing citrate later in Day 2 revealed platelet count of 28 × 10^9^/L. A CBC obtained in a collection containing heparin was unable to report a platelet count by the analyzer due to platelet clumping (Table 1). Peripheral smears of blood draw samples containing heparin and citrate both showed platelet clumping when observed under an oil microscope (Figures 2 and 3).

## 2. Discussion

Pseudothrombocytopenia (PTCP) is laboratory phenomenon that can occur in hospitalized patients, with approximately 0.1 to 0.2% being EDTA-dependent [1, 2]. The EDTA-dependent mechanism of PTCP occurs due to a conformational change of platelet surface glycoprotein IIb/IIIa (GPIIb/IIIa) caused by EDTA, which allows natural IgM or IgG auto-antibodies to bind to GPIIb/IIIa, leading to platelet agglutination. The phenomenon only occurs *in vitro* and has no known associated clinical significance [3]. However, known risk factors for PTCP due to platelet clumping include active viral infections, capillary or central line blood collection, autoimmune conditions such as rheumatoid arthritis, and medications, such as the GPIIb/IIIa inhibitor abciximab used for the treatment of acute coronary syndrome when undergoing percutaneous coronary intervention [4–7].

A high degree of suspicion is required. PTCP should be on the differential diagnosis for any patient presenting with moderate to severe thrombocytopenia on automated CBC but no clinical signs or symptoms of mucocutaneous bleeding, and a peripheral blood smear be obtained. Otherwise, additional costs and risks associated from further diagnostic testing and treatment for alternate causes of thrombocytopenia may occur. For example, a patient suspected of presenting with ITP may be treated with corticosteroids or even splenectomy, leading to potential iatrogenic injury.

Diagnosis is confirmed by obtaining a blood smear showing platelet clumps. However, PTCP can also be suspected with alerts from an automated hematology analyzer, and a blood smear is thus not always required. Cases of PTCP have been reported in the literature [1–9]. It should be noted that type 2B von Willebrand disease may also lead to platelet clumping that has been misdiagnosed as PTCP. However, the platelet clumps in type 2B von Willebrand disease are generally larger and with more variation in size [8, 10].

In our patient, the most likely precipitating cause of PTCP was a viral infection, given the clinical presentation suggesting acute gastroenteritis. The patient was discharged on Day 2 of her hospital stay in stable condition. However, she was unable to follow-up with us as an outpatient as she returned to her home country. Given the diagnosis of PTCP, oral dexamethasone, which was initially given for empiric treatment of ITP, was discontinued.

Interestingly, this rare laboratory phenomenon appeared to be highly unique in our patient as platelet clumping and pseudothrombocytopenia were observed not only with EDTA tubes, but also with tubes containing heparin and citrate (Table 1). The lack of resolution of platelet clumping in collection tubes containing either heparin or citrate suggests that heparin or citrate may also cause conformational changes to platelet surface glycoproteins in a similar mechanism as that of EDTA that allows binding of certain auto-antibodies. In such a case, a comprehensive work-up to identify alternate etiologies such as autoimmune etiologies is warranted.

Further confirmatory testing for PTCP can be performed by demonstration of a normal platelet count when repeating the CBC with a blood draw obtained in a collection tube containing citrate or heparin, the lack of platelet clumping on a repeat blood smear, or obtaining a peripheral blood film from a drop of blood from a finger stick showing no platelet clumping. The storage temperature of the collection tube has also been reported to affect the occurrence rate of PTCP. It has been reported the antiplatelet antibodies causing PTCP occur optimally between 0°C and 4°C, and that samples that are kept warm at approximately 37°C are less likely to experience clumping [11].

Prognosis of PTCP often involves full recovery when the underlying precipitating factor is treated, as described in several case reports [6, 12]. For example, in the case report by Hsieh et al. of a patient with PTCP associated with infectious mononucleosis, follow-up laboratory studies two months and 1 year after the initial presentation, after the patient had fully recovered from infectious mononucleosis, showed normalization of platelet counts and with no platelet clumping seen on peripheral blood smear [6]. While our patient was unable to follow-up with us, full recovery of PTCP would also be expected as the acute viral gastroenteritis resolved.

## Figures and Tables

**Table 1:** Patient's CBC and platelet values during hospital presentation.

Day and time of hospital visit	Platelet count (10^9^/L)	WBC (10^9^/L)	Hb (g/dL)	Additive in collection tube	Collection method
Day 1 at 9 : 33	11	10.1	15.2	EDTA	Venipuncture
Day 1 at 15 : 51	10	7.9	13.2	EDTA	Venipuncture
Day 2 at 6 : 58	10	5.0	12.4	EDTA	Venipuncture
Day 2 at 6 : 58	53	—	—	Citrate	Venipuncture
Day 2 at 6 : 58	Unable to report due to platelet clumping	—	—	Heparin	Venipuncture
Day 2 at 12 : 56	28	—	—	Citrate	Venipuncture
